# Modified regimen intrapleural alteplase with pulmozyme in pleural infection management: a tertiary teaching hospital experience

**DOI:** 10.1186/s12890-022-01995-z

**Published:** 2022-05-17

**Authors:** Xiong Khee Cheong, Andrea Yu-Lin Ban, Boon Hau Ng, Nik Nuratiqah Nik Abeed, Nik Azuan Nik Ismail, Nik Farhan Nik Fuad, Syed Zulkifli Syed Zakaria, Sheah Lin Ghan, Mohamed Faisal Abdul Hamid

**Affiliations:** 1 Respiratory Unit, Department of Medicine, Universiti Kebangsaan Malaysia Medical Centre, Jalan Yaacob Latif, Bandar Tun Razak, 56000 Kuala Lumpur, Malaysia; 2 Department of Radiology, Universiti Kebangsaan Malaysia Medical Centre, Kuala Lumpur, Malaysia; 3 Department of Paediatrics, Universiti Kebangsaan Malaysia Medical Centre, Kuala Lumpur, Malaysia; 4 Department of Pharmacy, Universiti Kebangsaan Malaysia Medical Centre, Kuala Lumpur, Malaysia

**Keywords:** Intrapleural fibrinolysis, Pleural infection, Empyema, Alteplase, DNase

## Abstract

**Background:**

Current management of poorly draining complex effusions favours less invasive image-guided placement of smaller tubes and adjunctive intrapleural fibrinolysis therapy (IPFT). In MIST-2 trial, intrapleural 10 mg alteplase (t-PA) with 5 mg of pulmozyme (DNase) twice daily for 72 h were used. We aimed to assess the effectiveness and safety of a modified regimen 16 mg t-PA with 5 mg of DNase administered over 24 h in the management of complex pleural infection.

**Methods:**

This was a single centre, prospective study involving patients with poorly drained pleural infection. Primary outcome was the change of pleural opacity on chest radiograph at day 7 compared to baseline. Secondary outcomes include volume of fluid drained, inflammatory markers improvement, surgical referral, length of hospitalisation, and adverse events.

**Results:**

Thirty patients were recruited. Majority, 27 (90%) patients were successfully treated. Improvement of pleural opacity on chest radiograph was observed from 36.9% [Interquartile range (IQR 21.8–54.9%)] to 18.1% (IQR 8.8–32.7%) of hemithorax (*P* < 0.05). T-PA/DNase increased fluid drainage from median of 45 mls (IQR 0–100) 24 h prior to intrapleural treatment to 1442 mls (IQR 905–2360) after 72 h; (*P* < 0.05) and reduction of C-reactive protein (*P* < 0.05). Pain requiring escalation of analgesia affected 20% patients and 9.9% experienced major adverse events. None required surgical intervention.

**Conclusion:**

This study suggests that a modified regimen 16 mg t-PA with 5 mg DNase can be safe and effective for patients with poorly drained complex pleural infection.

*Trial registration* The study was registered retrospectively on 07/06/2021 with ClinicalTrials number NCT04915586 (https://clinicaltrials.gov/ct2/show/NCT04915586).

**Supplementary Information:**

The online version contains supplementary material available at 10.1186/s12890-022-01995-z.

## Background

Pleural infection causes significant morbidity and mortality. Treatment goals involve antibiotic therapy and drainage of infected pleural fluid. However, unsatisfactory drainage can occur due to septations or loculated effusion. Surgery is indicated when medical therapy fails.

Recently, there is a paradigm shift to less invasive approach using intrapleural fibrinolytic therapy (IPFT) in complex effusion for patients who fail standard care and unfit for surgical intervention [1–3]. Intrapleural streptokinase and urokinase had been studied with conflicting results [4, 5]. Currently, tissue plasminogen activator (t-PA) e.g. alteplase is the most commonly used agent [6].

Pulmozyme (DNase) has a synergistic effect with alteplase on the breakdown of fibrin in empyema and reduces viscosity thus promoting pleural fluid drainage. Intrapleural combination t-PA and DNase success has been shown in numerous trials [7–12]. In the Multi-Centre Intrapleural Sepsis-2 (MIST-2) trial, the use of t-PA with DNase improved the drainage in patients with complex effusion and reduced the frequency of surgical referrals [7].

As far as our knowledge, there is no optimal regimen of intrapleural fibrinolysis therapy [13, 14]. The dosage that utilised in MIST-2 trial which 10 mg t-PA (with supplementary 5 mg DNase) twice daily is still an empiric choice [7]. Abu-Daff et al. shown the effectiveness of intrapleural 16 mg t-PA alone in complex effusion with 6.6% bleeding events reported [15].The rationale of using 16 mg t-PA in this study to simplify the pharmacy dispensary as in our formulary, one ampoule of alteplase contains 50 mg; t-PA biochemical stability up to 24 h following reconstitution as assessed by in vitro clot lysis assays [16]. We hypothesize that this modified regimen of t-PA 16 mg (with DNase 5 mg) is safe and effective for patients with poorly drained complicated pleural infection.

## Methods

We conducted a prospective open-label study on patients who were given intrapleural t-PA and t-PA for pleural infection from December 2019 to October 2020 in Universiti Kebangsaan Malaysia (UKM) Medical Centre. Written informed consents were obtained from all patients prior to participation in this study according to institutional guidelines.

### Patient involvement

We included patients with pleural infection (complex parapneumonic effusion or empyema) with poor pleural fluid drainage of ≤ 150 mL after 24 h of insertion of chest drain. Pleural infection was defined as fulfilled ≥ 2 of the following characteristics: 1) clinical evidence of infection such as fever and or elevated C-reactive protein (CRP) or white-cell count (WCC). 2) complex pleural effusion proven by thoracic ultrasound is defined as presence of fibrin strands or septations within pleural cavity. proven by thoracic ultrasound. 3) pleural fluid that fulfilled at least one of the characteristics: frank pus, exudative nature (according to light’s criteria), gram stain or culture positive, lactate dehydrogenase (LDH) > 1000 U/L, pH < 7.2 and/or glucose level < 3.3 mmol/L [7, 8]. The exclusion criteria were if candidates refused the trial, known allergy to t-PA or DNase, acute stroke, active bleeding diathesis, major surgery in past 5 days, previous pneumonectomy on the infected side, bronchopleural fistula, pregnancy or coagulopathy (INR > 2, APTT > 100, platelet count < 50,000 cells).

All patients underwent ultrasound guided intercostal chest catheter (ICC) placement. Baseline chest radiograph was performed within 24 h prior to IPFT to ensure the ICC position. The standard regime in our study was t-PA 16 mg with DNase 5 mg. Both medications were diluted in 50 mL of 0.9% sodium chloride solution respectively. T-PA 16 mg was instilled first intrapleurally via ICC, which was then allowed to dwell for 45 min and then unclamped to allow free drainage for 45 min. The same procedure was then repeated for DNase 5 mg. Therapy was given 12 h apart for a maximum of 3 doses, depending on physician’s judgement and clinical improvement. Intravenous tramadol 50 mg was administered prior to intrapleural alteplase as pre medication analgesia.

### Outcome measures

The primary outcome was the change in pleural opacity on hemithorax (measured in percentage), on chest radiograph at day 7 as compared to baseline. The pleural effusion volume was measured digitally (see Additional file 1) by two radiologists independently using Horos Project Software v3.3.5. [7, 17]. The radiologists were blinded to patient identity and intervention timing.

Secondary outcomes were the pleural fluid volume drained; the changes in inflammatory markers (CRP and WCC), length of hospitalisation, surgical intervention within 30 days and adverse events post therapy.

Treatment success defined as survival to hospital discharge without requiring surgical intervention within 30 days post first dose of intrapleural t-PA/DNase. Radiological improvement defined as reduction of pleural opacity ≥ 15% on day 7 chest radiograph compared to baseline. Treatment failure defined as failure of radiological resolution on chest radiograph, requirement of surgical intervention or mortality within 30 days post first dose of t-PA/DNase.

### Statistical analysis

The sample size was calculated on the basis of the estimated 95% confidence interval (95% CI) with population size taken from the previous study [18]. By using the formula with finite population correction (Daniel, 1999) with prevalence of 0.5 and precision of 5%, the sample size was 24.

Statistical analysis performed using SPSS software version 25 (IBM, Armonk, NY). All numerical data were subjected to normality testing using Kolmogorov–Smirnov test. Results presented as mean $\pm$ standard deviation (SD) or median (IQR) based on the normality of data. The Wilcoxon signed rank test were used to analyse the changes of pleural opacity on chest x-ray and inflammatory markers following intrapleural therapy. For multiple group comparisons analysis, we used Friedman’s analysis of variance on ranks (ANOVA), followed by post hoc test for pleural fluid drainage following intrapleural therapy. Significant was defined as *P* < 0.05.

### Ethical approval

The study was approved by the Research Ethics Committee board of UKM (FF-2020–008) and registered with Medical Research and Ethic Committee of the Ministry of Health Malaysia (NMRR-19–2940-51,404). The trial was registered retrospectively on 07/06/2021 with ClinicalTrials number NCT04915586. This was inadvertent and due to unawareness requirement on international registry. However, there were no protocol violation from when recruitment began to the registration of the trial. The authors confirm that all ongoing and related trials for this intervention are registered.

## Results

**Fig. 1:**
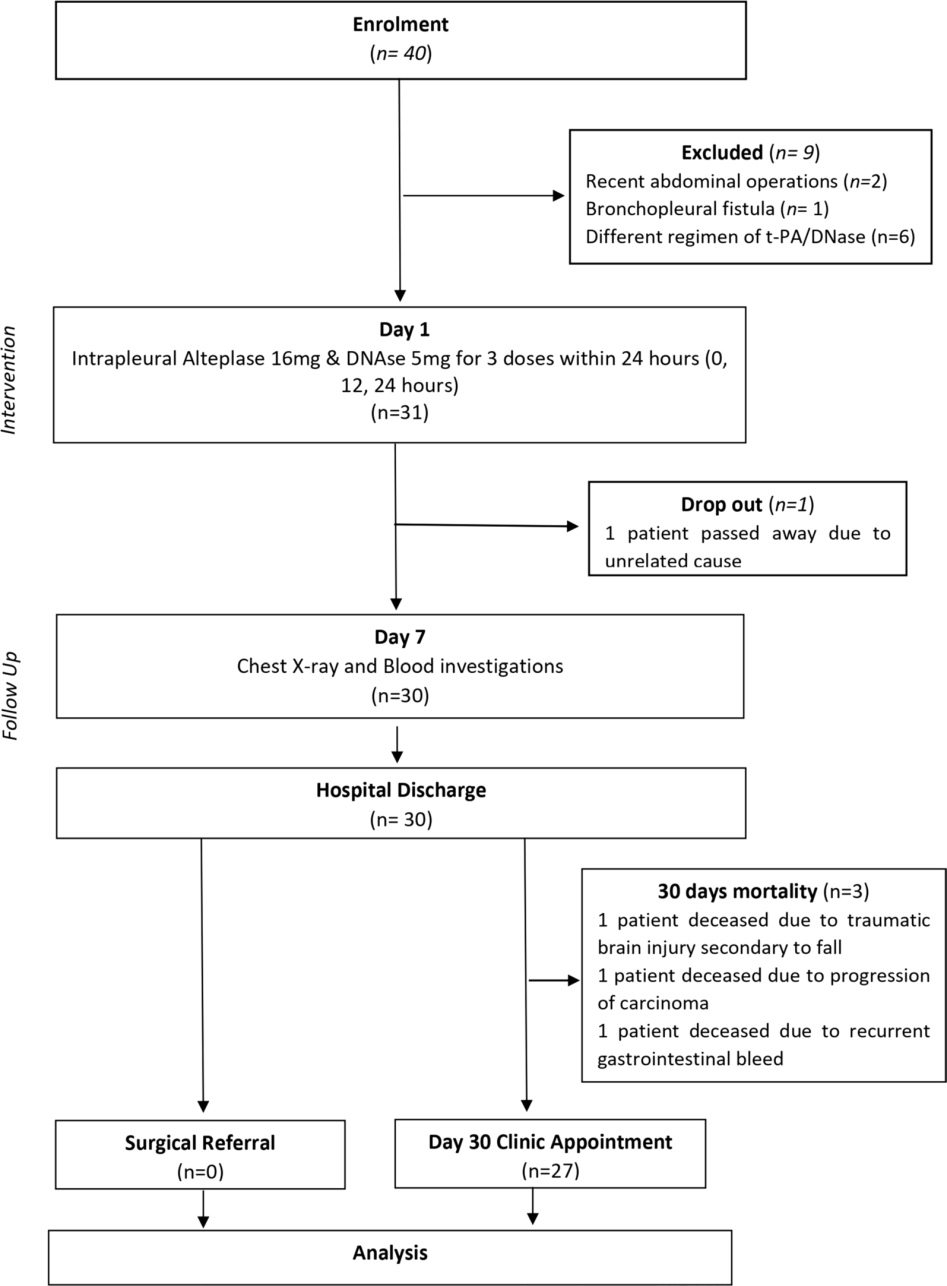
Study flowchart

Total 40 patients with pleural infection were enrolled during the study period, 9 patients were excluded due to the following reasons: 2–recent abdominal operations, 1-bronchopleural fistula and 6–different regimen of t-PA/DNase (Fig. 1). There was 1 drop out as patient passed away for myocardial infarction before completion of study. Demographic characteristics are detailed in Table 1. Total of 30 patients [23 males] with a mean age of 55.7 years (± SD 19) received intrapleural t-PA/DNase. Four patients (13.3%) had carcinoma as major life limiting illness. All 30 patients (100%) had septations on thoracic sonography. The median pleural fluid LDH was 1034 U/L (IQR 550–4395) (Table 1). Positive microbial pleural fluid cultures were reported in 8 (26.7%) patients; 4 patients (16.7%) grew *Klebsiella pneumoniae sp* (Table 2).

**Table 1:** Demographics and clinical characteristics

Demographic and characteristics	Values
Age (years), mean	55.7
Male sex, n (%)	23 (76.7)
Race, n (%)	
Malay	15 (50)
Chinese	13 (43.3)
Indian	2 (6.7)
Smoking History, n (%)	
Active smoker	9 (30)
Ex- smoker	9 (30)
Non smoker	12 (40)
Comorbidities, n (%)	30 (100)
Cardiovascular disease	
Hypertension	17 (56.7)
Ischemic heart disease	5 (16.7)
Atrial fibrillation	1 (3.6)
Congestive cardiac failure	2 (7.1)
Endocrine disease	
Diabetes mellitus	14 (46.7)
Respiratory disease	
Pulmonary tuberculosis (in the past)	2 (6.7)
Neurological disease	
Stroke	3 (10)
Renal disease	
Chronic kidney disease	3 (2 on dialysis) (10)
Malignancy	
Lung malignancy	2 (6.7)
Haematological malignancy	1 (3.3)
Retroperitoneal liposarcoma	1 (3.3)
Other	
HIV	2 (6.7)
Thalassemia major	1 (3.3)
Pleural infection characteristics	
Ultrasonographic evidence of septations, n (%)	30 (100)
Pleural fluid analysis	
Bacteria identified in pleural fluid culture, n (%)	8 (26.7)
Median LDH, U/L (IQR)	1034 (550–4395)
Median pleural fluid glucose, mmol/L (IQR)	4.9 (1.4–7.9)
Mean pleural fluid pH (SD)	8.0 ± 0.49
Intercostal catheter	
> 1 intercostal catheter, n (%)	11 (36.7)
Small bore intercostal catheter, ≤ 15 French, n (%)	26 (86.7)

*IQR* interquartile range, *SD* standard deviation, *LDH* lactate dehydrogenase, *ULN* upper limit of normal

**Table 2:** Microbiology in pleural fluid cultures

Microorganism cultured in pleural fluid	N (%)
**Negative cultures**	**22 (73.3)**
**Positive pleural fluid cultures**	**8 (26.7)**
*Gram positive organism*	*1 (3.3)*
Rhodococcus equi	1 (3.3)
*Gram negative organism*	*6 (23.4)*
Klebsiella pneumoniae sp.	4 (16.7)
Klebsiella ESBL	1 (3.3)
Enterobacter CRE	1 (3.3)

*ESBL* extended spectrum beta lactamase, *CRE* carbapenem resistance enterobacteriaceae

The median pleural fluid volume drained 24 h prior to intrapleural therapy was 45 ml (IQR, 0–100). Majority (86.7%) were treated with small bore (≤ 15 French) ICC. Eleven patients (36.7%) had more than 1 ICC inserted due to non–communicating locules of the effusion or chest drain dysfunction. Only 26.7% [8 patients] received ≤ 2 doses t-PA/DNase as effusion was completely drained before 3rd dose.

All 30 patients (100%) survived upon hospital discharge without requiring surgery (Table 3). No mortality due to pleural infection or directly related to t-PA/DNase therapy during 30 days after hospital discharge. Three patients died after hospital discharge within 30 days following intrapleural therapy due to unrelated causes as their pleural infection had been controlled. One patient died 20 days after discharge due to progression of lung carcinoma and poor comorbidities; while another patient with advanced peritoneal liposarcoma with colon metastasis had recurrent gastrointestinal bleed 7 days following intrapleural t-PA/DNase. One patient was on dabigatran for atrial fibrillation; died 15 days after discharge due to traumatic brain injury following fall at home. Therefore, 90% (27 patients) were successfully treated and alive at 30 days without requiring surgical intervention.

**Table 3:** Clinical outcomes

Clinical outcomes	Values
Treatment success, n, %	27 (90)
Surgical referral, n, %	0 (0)
Survival upon hospital discharge, n, %	
Mortality at 30 days, n, %	30 (100)
	3 (10)
Median length of hospital stay, days (IQR)	17 (11.7–24.2)
Median length of stay from first dose intrapleural therapy, days (IQR)	
Median duration of intercostal catheter in pleural cavity, days (IQR)	8 (4.8–12)
Median duration of intercostal catheter in pleural cavity post first dose intrapleural therapy, days (IQR)	
Patients who received ≤ 2 doses intrapleural therapy, n (%)	10 (8–14.3)
	6 (4–7.3)
	8 (26.7)
**Adverse events**^**a**^**, n, %**	
Chest pain requiring escalation of analgesics	6 (20)
Gastrointestinal bleed	1 (3.3)
Clinical deterioration	1 (3.3)
Hemoptysis	1 (3.3)

*IQR* interquartile range, *SD* standard deviation, *CRP* C-reactive protein, *WBC* white cell count

^a^Same patient might experienced more than one adverse events

The median length of hospital stay were 17 days (IQR, 11.7–24.2) (Table 3). The median duration of hospitalisation from first dose post intrapleural therapy were 8 days (IQR, 4.8–12).

**Fig. 2:**
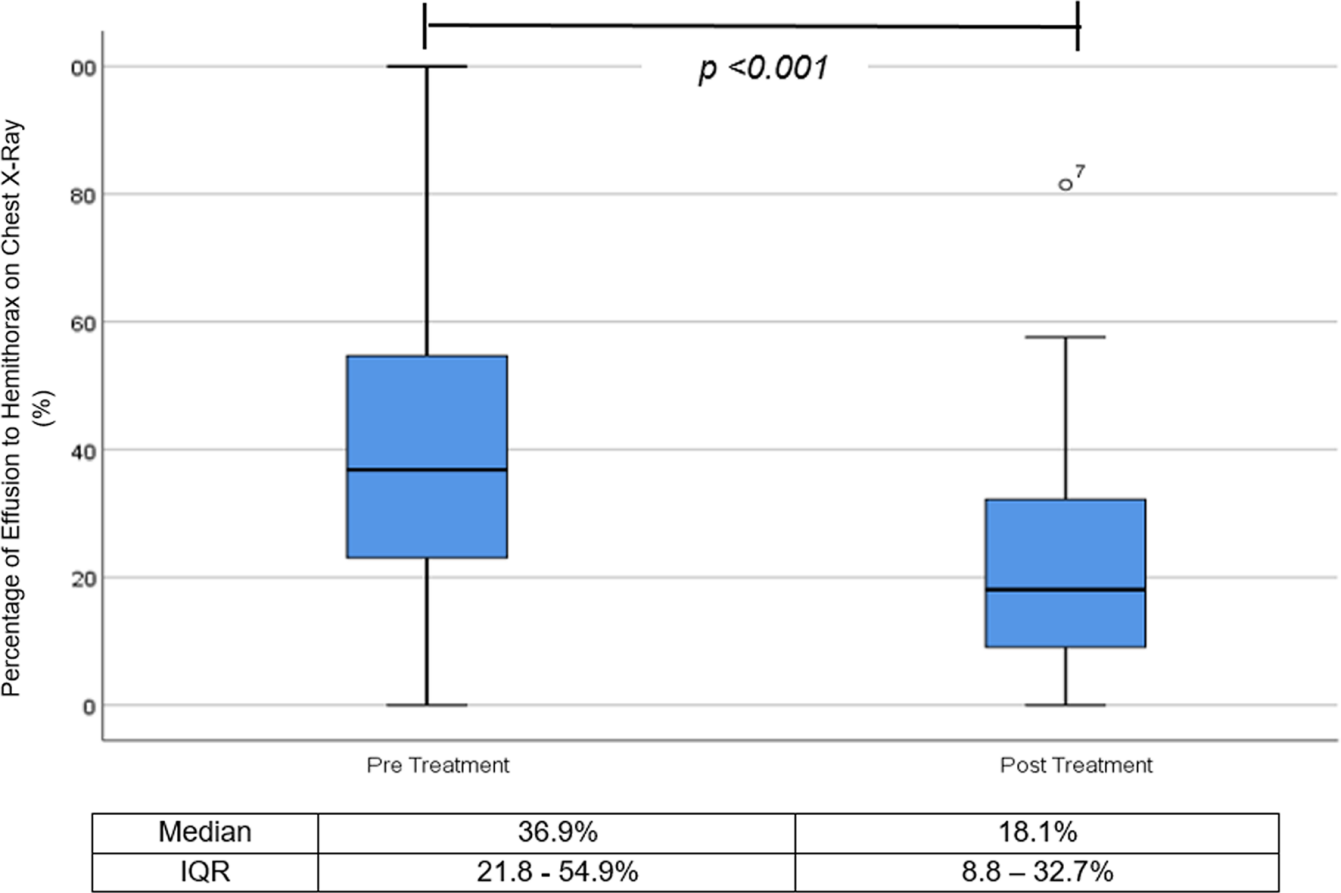
Change in pleural effusion on chest radiograph (*n* = *30*), measured in percentage of the pleural opacity on hemithorax before intrapleural t-PA/DNase (pre-treatment) and day 7 following first dose t-PA/DNase (post treatment). One patient was excluded from analysis due to passed away for unrelated cause prior to day 7. *P* < 0.05 by Wilcoxon signed-rank test. *IQR* interquartile range

Significant radiological improvement was observed with intrapleural t-PA/DNase (Fig. 2). Pleural opacity on chest x-ray (CXR) was reduced from a median of 36.9% (IQR, 21.8–54.9) on baseline chest x-ray to median of 18.1% (IQR, 8.8–32.7) after 7 days post intrapleural therapy (*P* < 0.001).

**Fig. 3:**
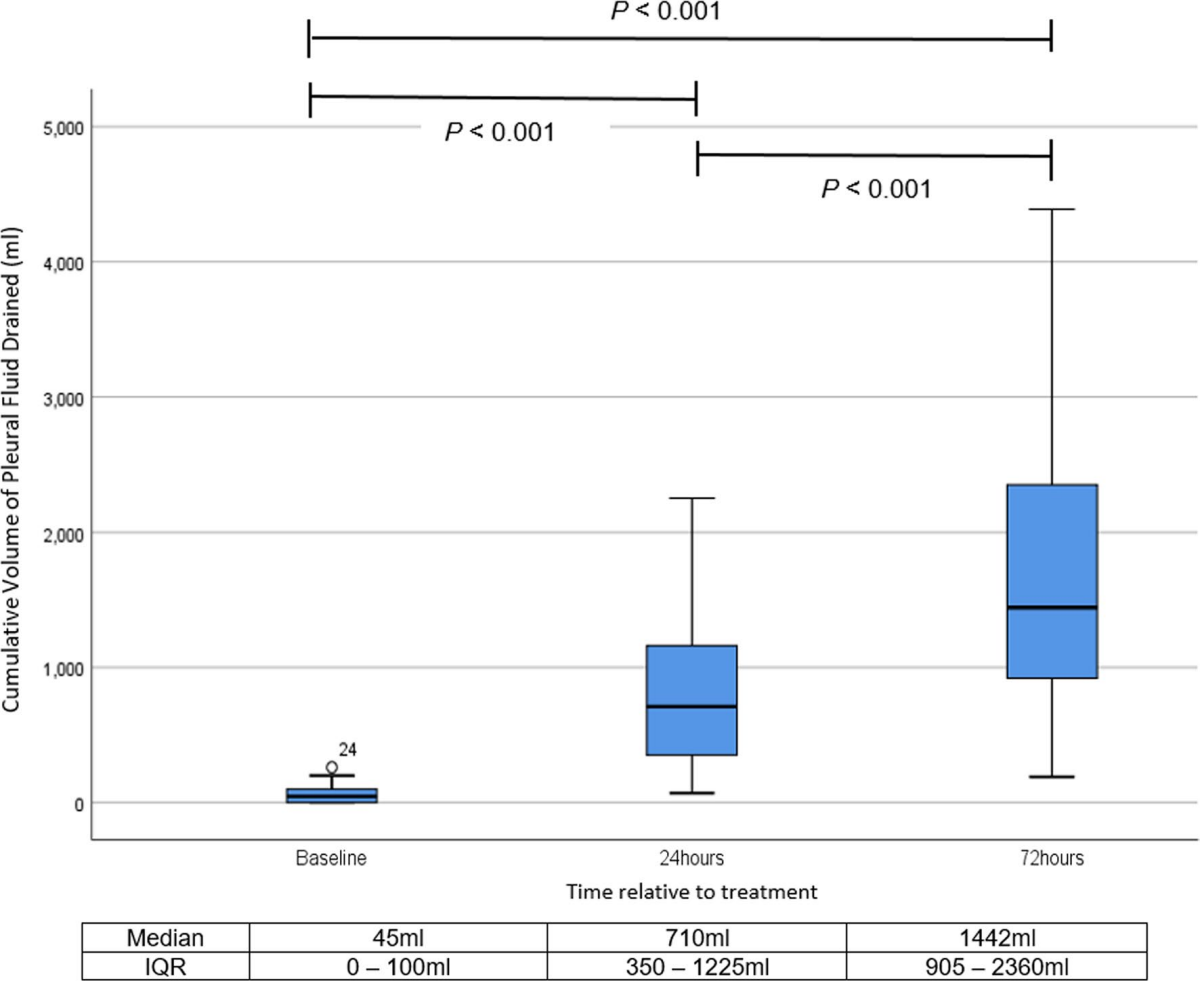
Cumulative volume of pleural fluid drained (n = 30) in the 24 h before treatment (baseline), at 24 h and 72 h post first dose of intrapleural t-PA/DNase. *P* < 0.05 by Friedman’s ANOVA followed by post hoc test. *IQR* interquartile range

The median (IQR) pleural fluid drainage 24 h before t-PA/DNase therapy was 45 ml (0–100), which increased to 710 ml (350–1225) at 24 h following the first dose t-PA/DNase and 1442 ml (905–2360 at 72 h of therapy (*P* < 0.001) (Fig. 3).

There was significant reduction on CRP (normal range < 0.5 mg/dL) with median (IQR) at baseline pre-treatment of 11.4 mg/dL (4.5–19.4) to 5 mg/dL (1.8–10.4 mg/dL) by day 7 post intrapleural t-PA/DNase (*P* < 0.001). However, there was no significant change in the median (IQR) on day 7 post therapy WCC of 7.9 × 10^9^ (6.1–11.0) from baseline 8.2 × 10^9^ (7.1–13.1) (*P* = 0.215).

Three major adverse events (9.9%) were reported (Table 3); 2 events seen in one patient which include gastrointestinal (GI) bleed (3.3%) and hypotension (3.3%) while another patient had mild hemoptysis (3.3%). First patient had bleeding risk and advanced peritoneal liposarcoma with colon metastasis; not suitable for surgery. He developed GI bleed following first dose intrapleural t-PA/DNase which the treatment was ceased. His condition improved with blood transfusion and bleeder secured; sepsis was controlled with radiological improvement. However, GI bleed recurred 7 days after intrapleural t-PA/DNase was given and he passed away subsequently.

Second patient had one episode of hemoptysis (3.3%) after second dose t-PA/DNase. He was on dialysis for end stage renal disease. However, no evidence of pleural bleeding and patient remained hemodynamic stable throughout therapy.

Another patient had recent chemotherapy for diffuse B cell lymphoma. Total 2810 ml blood-stained pleural fluid was drained after three doses of t-PA/DNase. Her haemoglobin level gradually decreased (8.6 g/L to 7.8 g/L) without hemodynamic compromise. She was transfused one unit packed cell and was discharged well.

Six (20%) cases had chest pain within first 12 h following intrapleural therapy. All patients responded well to escalation of opioid analgesia without cessation of treatment.

## Discussion

Majority of patients (90%) were successfully treated and none required surgery for pleural infection. The clinical success was corroborated by significant improvements in radiographic clearance and pleural fluid drainage. This study demonstrated a comparable success rate to previous studies [7, 8]. Comparison of clinical outcomes between studies should be made with caution due to the differences in patients’ cohorts, clinical populations and study methodologies.

Majority (19.9%) had major life limiting diseases (carcinoma and end stage renal disease) who were unfit for surgery. Our baseline data showed our study cohort had significant pleural infection, as evidenced by high LDH, positive pleural fluid culture or septated effusion visualised by thoracic ultrasonography. Pleural fluid pH analysis may be inaccurate in this study due to our samples were not transported in heparinised blood gas syringe or measured in blood gas analyser. It was measured with litmus paper.

A longer hospital stay observed in our study as our cohort mainly those with poor drainage and failed medical therapy; in contrast with MIST-2 trial, intrapleural therapy were given immediately after randomisation. Concurrent bacteremia or other organ abscess that required longer parenteral antibiotics therapy also impact the length of stay. Furthermore, we do not have outpatient parenteral antimicrobial therapy service and these patients had to stay to complete the intended duration of parenteral antibiotics.

Dosing of t-PA used for intrapleural fibrinolysis varies from 4 to 100 mg per day [7, 14, 19–21], while the dose of intrapleural alteplase (t-PA) used in MIST-2 at 10 mg with supplemental pulmozyme (DNase) 5 mg twice daily for six doses is still empiric choice [7]. The rationale of this modified regimen of 16 mg t-PA with 5 mg DNase for total 3 doses that administered sequentially within 24 h reduced the frequency and time consuming for health care provider on administration of the drugs.

We propose that this modified regimen of t-PA and DNase offer an alternative therapeutic option for patients that are unfit or refuse surgical intervention but persistent pleural infection. We have demonstrated similar treatment success comparable to other studies [7, 8, 12]; as evidenced by improvement on pleural fluid drainage and reduction in pleural opacity on day 7 chest x-ray was approximately 50% from the baseline using intrapleural 16 mg t-PA with 5 mg DNase. The mechanism of action of t-PA and DNase in pleural cavity remain unclear. Studies suggested that IPFT may trigger the monocyte chemoattractant protein 1 (MCP-1) pathway which promote pleural fluid formation and subsequently causes a therapeutic lavage effect that increases pleural fluid drainage [22, 23].

Majority of our population (86.7%) was treated with small borne intercostal catheter (≤ 15 French) as small bore chest drains have been shown experienced less pain than the larger one [24].

Bleeding risk has been a concern with intrapleural fibrinolysis although the mechanism of action t-PA and DNase in pleural space still unknown. Reported major adverse events (9.9%) in this study was slightly higher than previous studies [7, 8]. Both patients had underlying comorbidities (advanced carcinoma and end stage renal disease) might contribute to the occurrence of bleeding event and clinical deterioration rather than purely due to the side effects of fibrinolytic agent. The contribution of t-PA or DNase to the bleeding event in our patient is uncertain as t-PA has short systemic half-life of 4–6 min and his GI bleed recurred 7 days after stopped intrapleural t-PA/DNase.

Chest pain was the main adverse effect, 20% patients requiring escalation of analgesia similar to previous studies that using conventional regimen [7, 8, 25].

There were some limitations to our study. It was a single centre, prospective one-arm study. Thus, there is some difficulty in interpreting the results due to absence of control group. A multicentre randomised, placebo-controlled study should be conducted to assess the efficacy and safety of the optimal dose of combined t-PA/DNase in treatment of pleural infection due to small number of cases seen in a single institution. Furthermore, the criteria to perform surgery or insert an additional drain were not clearly protocoled; treatment decisions were made by the physicians which may have led to heterogeneity in management.

## Conclusion

This short duration modified regimen intrapleural 16 mg t-PA with 5 mg DNase is safe and effective in the management of poorly drained pleural infection. Administration of IPFT can be a rescue therapy for those who unfit for surgery with persistent pleural infection. In future, a large randomised placebo-controlled study could be conducted to evaluate the effectiveness and safety of different dosing regimen of intrapleural t-PA/DNase.

## Supplementary Information

**Additional file 1:** Digital measurement on pleural opacity on chest X-ray.

## Data Availability

The datasets used and/or analysed during the current study available from the corresponding author on reasonable request.

## Abbreviations

IPFT: Intrapleural fibrinolysis therapy
t-PA: Alteplase
DNase: Pulmozyme
IQR: Interquartile range
SD: Standard deviation
LDH: Lactate dehydrogenase
ULN: Upper limit of normal
ESBL: Extended Spectrum Beta Lactamase
CRE: Carbapenem resistance Enterobacteriaceae
