# Correction: Modified regimen intrapleural alteplase with pulmozyme in pleural infection management: a tertiary teaching hospital experience

**DOI:** 10.1186/s12890-022-02251-0

**Published:** 2022-11-23

**Authors:** Xiong Khee Cheong, Andrea Yu-Lin Ban, Boon Hau Ng, Nik Nuratiqah Nik Abeed, Nik Azuan Nik Ismail, Nik Farhan Nik Fuad, Syed Zulkifli Syed Zakaria, Sheah Lin Ghan, Mohamed Faisal Abdul Hamid

**Affiliations:** 1 Respiratory Unit, Department of Medicine, Universiti Kebangsaan Malaysia Medical Centre, Jalan Yaacob Latif, Bandar Tun Razak, 56000 Kuala Lumpur, Malaysia; 2 Department of Radiology, Universiti Kebangsaan Malaysia Medical Centre, Kuala Lumpur, Malaysia; 3 Department of Paediatrics, Universiti Kebangsaan Malaysia Medical Centre, Kuala Lumpur, Malaysia; 4 Department of Pharmacy, Universiti Kebangsaan Malaysia Medical Centre, Kuala Lumpur, Malaysia

## Correction: BMC Pulmonary Medicine (2022) 22:199 10.1186/s12890-022-01995-z

Following publication of the original article [1], the authors flagged that some of the values in Table 3 were incorrectly aligned. The table has now been corrected in the published article. Please be referred to the (now correctly aligned) values in the corrected table.
