# Supplementary material for: Modified regimen intrapleural alteplase with pulmozyme in pleural infection management: a tertiary teaching hospital experience
Source: BMC Pulm Med. 2022 May 17;22:199. doi: 10.1186/s12890-022-01995-z (PMC9115979; doi:10.1186/s12890-022-01995-z)
Supplement: Supplementary file 1 — Additional file 1: Digital measurement on pleural opacity on chest X-ray. [file 12890_2022_1995_MOESM1_ESM.pdf]

Digital measurement on pleural opacity on Chest X-ray:

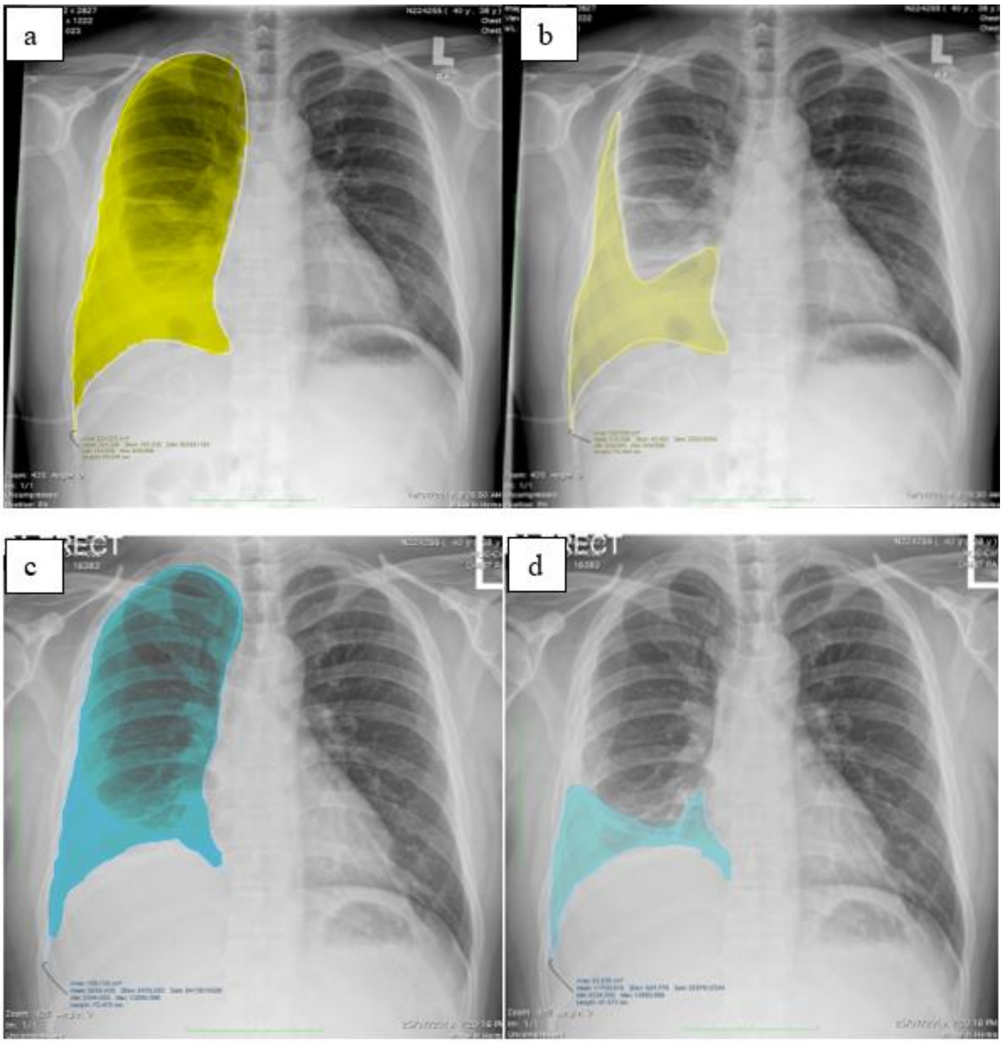

The hemithorax areas were on day1 and day7 as shown in figure a & c. The area of pleural opacity is shown at baseline (day 1) in figure b and at day 7 in figure d. The percentage of the hemithorax area occupied by pleural opacity on day 1 was calculated as the yellow coloured area in figure b divided by that in figure a and then multiplied by 100. The corresponding percentage on day 7 was calculated by dividing the blue coloured area in figure d by that in figure c and multiplying by 100. The change in the percentage of hemithorax area occupied by pleural opacity (the primary outcome) between day 7 and day 1 was calculated.
